# Association between COVID-19 anxiety syndrome and COVID-19 vaccine hesitancy in the postpandemic era: a cross-sectional study in Hong Kong

**DOI:** 10.1186/s12889-025-21367-6

**Published:** 2025-01-14

**Authors:** Timothy Chung Ming Wu, Jonathan Ka Ming Ho, Sai Kit Choi, Yanki Hiu Yan Chan, Bella Wing Sze Chan, Timmy Tim Ming Li, Fu Po Tam, Ivan Man Chun Wong, Alex Siu Wing Chan

**Affiliations:** 1 School of Nursing and Health Sciences, Hong Kong Metropolitan University, Hong Kong, China; 2 Institutes of Brain Science, Fudan University, Shanghai, China; 3 Department of Medicine, Queen Mary Hospital, Hospital Authority, Hong Kong, China; 4 Department of Neurosurgery, Prince of Wales Hospital, Hospital Authority, Hong Kong, China; 5 Department of Medicine, Pamela Youde Nethersole Eastern Hospital, Hospital Authority, Hong Kong, China; 6 Department of Operating Theatre, Union Hospital, Hong Kong, China; 7 Department of Medicine and Geriatrics, Pok Oi Hospital, Hospital Authority, Hong Kong, China; 8 Department of Social Work, Hong Kong Baptist University, Hong Kong, China

**Keywords:** COVID-19 anxiety syndrome, COVID-19 vaccine hesitancy, Maladaptive coping, Avoidance behaviour, Fear, Threat

## Abstract

**Background:**

The COVID-19 pandemic has had profound psychophysiological and socioeconomic effects worldwide. COVID-19 anxiety syndrome (CAS) is a specific cluster of maladaptive coping strategies, including perseveration and avoidance behaviours, in response to the perceived threat and fear of COVID-19. CAS is distinct from general COVID-19 anxiety. The level of CAS in the postpandemic era remained unknown. Despite extensive research on general COVID-19 anxiety and COVID-19 vaccine hesitancy (CVH), few studies have investigated the association between CAS and CVH. The present study aimed to assess the level of CAS and the prevalence of CVH and explore the association between CAS and CVH in the general population of Hong Kong.

**Methods:**

This cross-sectional study was conducted in Hong Kong. Participants were recruited using convenience and snowball sampling methods and completed an online or a paper-based questionnaire comprising two well-validated instruments. The COVID-19 Anxiety Syndrome Scale (C-19ASS), which includes the C-19ASS-P and C-19ASS-A subscales, was used to evaluate CAS in terms of perseveration and avoidance behaviours. The COVID-19 Vaccine Hesitancy Scale (CVHS) was used to determine the presence of CVH.

**Results:**

This study included 389 participants. The median C-19ASS-P and C-19ASS-A scores were 8 (Interquartile range (IQR) 5–13) and 3 (IQR 0–6), respectively. The CVHS scores revealed a CVH prevalence of 68.1%. A significantly larger proportion of participants with CVH rated “hesitant” compared with those without CVH across all the CVHS items. Furthermore, the median C-19ASS-P and C-19ASS-A scores were significantly higher for participants without CVH than for those with CVH.

**Conclusions:**

Our findings revealed that CAS persists and CVH is common in the postpandemic era and that CAS is associated with CVH. Comprehensive interventions addressing both informational and psychological aspects are needed to increase the rate of vaccine acceptance and to mitigate the effect of CAS on public health outcomes.

**Supplementary Information:**

The online version contains supplementary material available at 10.1186/s12889-025-21367-6.

## Introduction

After its outbreak in Wuhan, China in December 2019, COVID-19 rapidly evolved into a global pandemic, imposing extensive morbidity and mortality burdens [1]. As of February 18, 2024, 774,699,366 cases of COVID-19 and 7,033,430 deaths due to COVID-19 have been reported worldwide [2]. COVID-19 spread rapidly, overwhelmed healthcare systems, had profound socioeconomic effects, and prompted stringent public health measures in many countries [3]. Furthermore, the pandemic disrupted social interactions and led to widespread isolation and loneliness, and the closure of educational institutions [4, 5].

The COVID-19 pandemic affected both the physical health and mental health of individuals [6]. A recent systematic review of 43 studies revealed that there was a linear correlation between the exacerbation of depression and anxiety and the spread of COVID-19 (increasing case count) [7]. Another systematic review of 19 studies reported elevated prevalence of anxiety symptoms (6.33–50.9%), depression (14.6–48.3%), psychological distress (34.4–38%), and stress (8.1–81.9%) in the general populations of China, Spain, Italy, Iran, Turkey, Denmark, and the United States during the pandemic [8].

COVID-19 anxiety syndrome (CAS) is different from general COVID-19 anxiety. CAS is a specific cluster of maladaptive coping strategies, including perseveration and avoidance behaviours, in response to the perceived threat and fear of COVID-19 [1, 9, 10]. These maladaptive coping strategies prevent individuals from returning to normal daily activities and functioning, resulting in persisting psychological distress [9, 11, 12]. Information on the persistence of CAS beyond the acute phase of the pandemic is essential for comprehensively addressing COVID-19-related mental health problems [10].

Within a year after the onset of the COVID-19 pandemic, vaccines were developed to control the spread of the virus and mitigate the effects of the virus on society and healthcare system [13, 14]. Despite the widespread availability of COVID-19 vaccines and their recognition as an effective measure for controlling the pandemic, COVID-19 vaccine hesitancy (CVH)-a delay in acceptance or refusal to accept a COVID-19 vaccine [15]-emerged as a major global challenge [14, 16]. Recognising the importance of vaccination in pandemic control, researchers and healthcare professionals worldwide explored factors contributing to CVH [17, 18]. However, the measurement tools (i.e., questionnaires) they used for data collection varied widely in terms of scope, depth, and rating scales [19–25]. A personalised systematic and rigorous approach for assessing CVH is needed to promote collaborative efforts in unraveling its complexity [14].

Although the relationship between general COVID-19 anxiety and CVH has been previously explored, few studies have specifically investigated the association between CAS and CVH [26]. An association has been identified between COVID-19-related anxiety (or fear) and CVH. Individuals with higher levels of anxiety (or fear) were more likely to accept vaccination [26–28]. By contrast, individuals with lower levels of fear exhibited higher levels of CVH [29]. However, this topic remains open to investigation, given that anxiety and fear alone might not reliably predict CVH [30]. Drawing from the literature and the strong correlation between COVID-19-related psychological distress and CAS [9], we hypothesized that there is an association between CAS and CVH.

This study evaluated CAS level, estimated CVH prevalence, and identified the association between CAS and CVH in the general population. Limited information is available on the level of CAS in the general population. Moreover, the prevalence of CVH is unclear. By revealing the association between CAS and CVH, our study offers valuable insights into culturally sensitive approaches for mitigating CVH. At the time of the data collection of this study, the Hong Kong government had relaxed public health measures and commenced mass COVID-19 vaccination programmes. Thus, this study was a timely, and maybe the first, endeavour for assessing the persistence of CAS and for measuring the level of CAS beyond the acute stage of the COVID-19 pandemic.

## Methods

### Study Design

This study had a cross-sectional design. The findings are reported following the Strengthening the Reporting of Observational Studies in Epidemiology (STROBE) checklist for cross-sectional studies.

### Participants and sampling

Given the timeliness and feasibility of this study, convenience sampling and snowball sampling were adopted to recruit participants from the general population of Hong Kong. Target participants were recruited through invitations posted on various social media platforms, namely Facebook, WhatsApp, Signal, WeChat, and Instagram. Respondents were encouraged to share the online survey with their family members, friends, and co-workers. Individuals were included if they were (1) residing in Hong Kong, (2) aged 18 or above, and (3) able to speak and understand English or Chinese. Individuals were excluded if they were (1) not residing in Hong Kong within the previous two weeks, (2) recently vaccinated through the government’s COVID-19 vaccine programme, and (3) deemed mentally incompetent. Recognising that only 68.1% of the older population of Hong Kong owned their smartphones and acknowledging potential accessibility issues with social media platforms, we additionally conducted paper-based surveys at the entrances of various Mass Transit Railway stations [31].

The sample size was calculated using the following formula:

Sample size = $$\:{{(Z}_{1-\alpha\:/2})}^{2}$$ × P(1 − P)/$$\:{d}^{2}$$

where $\:{Z}_{1-\alpha\:/2}$ represents the standard normal variate (1.96 at the 95% confidence interval); *P* represents the expected proportion of individuals with CVH (set at 50% to maximize the sample size); and *d* represents the absolute precision (set at 5%; deemed acceptable in most studies). The required sample size calculated using these parameters was approximately 384 [32].

### Measures

Data were collected using a questionnaire comprising a demographic information form and two validated, structured instruments: the COVID-19 Anxiety Syndrome Scale (C-19ASS) and the COVID-19 Vaccine Hesitancy Scale (CVHS). The participants received either the Chinese or English version of the questionnaire.

### Demographic information form

A self-developed demographic information form was used to collect data on personal characteristics, namely sex, age, marital status, educational level, employment status, monthly household income, COVID-19 vaccine dose(s) received, and vaccine type.

### COVID-19 anxiety syndrome scale

English and Chinese versions of the 9-item C-19ASS were used to assess maladaptive coping strategies in response to COVID-19-related fear and anxiety [9]. The English version of the C-19ASS was developed by Nikčević and Spada [9]. This scale comprises two subscales: C-19ASS-P, which assesses perseveration, and C-19ASS-A, which assesses avoidance. Perseveration refers to behaviours such as checking the symptoms of COVID-19, worrying, and monitoring threats in response to the threat and fear of COVID-19. Avoidance refers to avoiding places that pose a high risk of COVID-19 transmission [9, 33]. The C-19ASS-P subscale comprised six items, and the C-19ASS-A subscale comprised three items. Responses were rated on a 5-point Likert scale with endpoints ranging from 0 (not at all) to 4 (nearly every day over the last 2 weeks). The total scores on C-19ASS-P and C-19ASS-A ranged from 0 to 24 and from 0 to 12, respectively. A higher subscale total score indicated a higher level of perseveration or avoidance in response to the threat or fear of COVID-19. In the original study, Cronbach’s alpha coefficients for the English versions of the C-19ASS-P and C-19ASS-A were 0.86 and 0.77, respectively, indicating good internal consistency [9, 34]. In a subsequent study, the English versions of the C-19ASS-P and C-19ASS-A exhibited good structural and convergent validity [11].

No traditional Chinese version of the C-19ASS was available. The simplified Chinese version of the C-19ASS was translated from the English version in the study of Xin et al. [35] and exhibits satisfactory internal consistency for the main scale (Cronbach’s alpha coefficient: 0.84) [34]. However, this version remains to be separately validated for the C-19ASS-P and C-19ASS-A subscales, as recommended by the original study [9]. Moreover, researchers doubt the validity of the simplified Chinese version of the C-19ASS. Thus, the original English version of the C-19ASS was translated into traditional Chinese by an expert in English-Chinese translation in this study. An expert panel then reviewed the translated text for contextual accuracy and validity. Subsequently, another translator proficient in both English and Chinese back-translated the traditional Chinese version into English. A third translator validated both versions and identified any discrepancies. On the basis of their feedback, amendments were made to the traditional Chinese version of the C-19ASS to ensure alignment with the original English version.

In this study, an expert panel comprising two health science professors, two physicians, and two registered nurses validated the newly developed traditional Chinese version of the C-19ASS to evaluate its cultural appropriateness and validity. The experts determined the content validity index (CVI) of the scale by independently rating each item for its relevance to Chinese culture on a 4-point Likert scale with endpoints ranging from 1 (*not relevant*) to 4 (*highly relevant and succinct*). The CVI represents the total percentage of items rated by experts as either 3 or 4. A CVI of $\:\ge\:$ 0.70 was deemed acceptable [34]. The traditional Chinese version of the C-19ASS achieved a CVI of 0.74, indicating satisfactory content validity. Before data collection, Cronbach’s alpha was used to assess the internal consistency for the traditional Chinese versions of both C-19ASS-P and C-19ASS-A in 53 Hong Kong residents who were aged 18 or above and able to speak and understand Chinese. Cronbach’s alpha coefficients for the two subscales were 0.86 and 0.88, respectively, indicating good internal consistency [34].

### COVID-19 vaccine hesitancy scale

English and Chinese versions of the 15-item CVHS were used to assess beliefs and attitudes towards CVH and reasons for vaccine refusal [36, 37]. The English version of the CVHS was developed by Huang et al. [36] and translated into traditional Chinese in the study of Huang et al. [37]. In the aforementioned studies, responses were rated on a 3-point Likert scale with endpoints ranging from 0 (*non-hesitant*) to 2 (*hesitant*). The total score (range: 0 to 30) was calculated by summing the scores on corresponding items. Subsequently, the total score was linearly transformed to a score ranging from 0 to 100 to account for items with missing value. A transformed score of ≥ 50 indicated the presence of CVH, while a transformed score of < 50 indicated its absence. The specific items and scoring rules for this scale can be found in Additional File 1. The specific item Cronbach’s alpha coefficients for the English and Chinese versions of the CVHS were 0.755 and 0.756, respectively, indicating good internal consistency [36, 37]. In addition, both versions exhibited satisfactory convergent, concurrent, and discriminant validity in determining the presence of CVH [37].

### Data Collection

Data were collected through in-person (self-administered paper-based questionnaires) or online (Google Form) surveys. At the time of data collection, local anti-epidemic measures in Hong Kong had been adjusted as follows: cessation of quarantine order issuance, relaxation of social distancing measures except mask-wearing, and withdrawal of the imposed criteria defining close contacts. The data collection period was opportune for determining whether CAS persists in the postpandemic era.

### Data analyses

Data were analysed using Statistical Package for the Social Science (SPSS; version 26; IBM Corporation, Armonk, NY, USA). CAS levels were assessed for normality using the one-sample Kolmogorov–Smirnov test. Descriptive statistics, such as frequency, percentage, median, and interquartile range (IQR), were used to describe participant personal characteristics, CVH prevalence, and CAS levels (perseveration and avoidance). The Pearson’s chi-square test was performed to compare the CVHS score between participants with CVH and those without CVH. A Mann–Whitney U test was performed to identify the association between CVH and CAS. All tests were two-tailed, and statistical significance was set as *p* ≤ 0.05.

### Ethical consideration

This study was approved by the Research Ethics Committee of the School of Nursing and Health Studies at the Hong Kong Metropolitan University. The study procedures followed the ethical principles of the Declaration of Helsinki, the Hong Kong Personal Data (Privacy) Ordinance, and institutional policies. All participants received an information sheet detailing the study’s purpose, procedures, potential benefits and risks, and participant rights. In the online survey, this information sheet was presented at the beginning to ensure that all participants were adequately informed. Written informed consent was obtained from participants completing the paper-based survey, whereas consent was assumed for those completing the online survey. Data were anonymous, maintained with strict confidentiality, and used for research purposes only.

## Results

### Personal characteristics

A total of 484 participants completed the 15-min online or paper-based surveys. After initial screening and data cleaning, 389 responses (online survey: 241 [62%]; paper-based survey; 148 [38%]) were deemed valid. Table 1 presents the personal characteristics of the participants (*N* = 389). The participant pool comprised almost equal proportions of men (49.9%) and women (50.1%). Of the participants, approximately 54.5% were aged 18–35 years. Nearly 55.0% of the participants were single, with the remainder being married (38.8%), divorced (2.6%), or widowed (3.6%). About 80% of the participants were educated at a tertiary (59.9%) or secondary (23.4%) level. Around 49.1% of the participants were employed full-time, 6.4% were housewives, and 21.1% were students. Close to 42.4% of the participants reported a monthly household income of > USD$3,834, which exceeds the 2022 Hong Kong median monthly household income (USD$3,616) [38]. Nearly all the participants (98.7%) had received a COVID-19 vaccine, with 70.7% having received three doses. Of the 384 participants who received the COVID-19 vaccine, 71.1% received the BioNTech vaccine, and 28.6% received the Sinovac vaccine.

**Table 1:** Personal characteristics of the participants (*N* = 389)

Personal characteristics	*n* (%)
**Gender**	
Male Female	194 (49.9) 195 (50.1)
**Age group (years)**	
18–25 26–35 36–45 46–55 56–65 Above 65	149 (38.3) 63 (16.2) 54 (13.9) 44 (11.3) 34 (8.7) 45 (11.6)
**Marital status**	
Single Married Divorced Widowed	214 (55.0) 151 (38.8) 10 (2.6) 14 (3.6)
**Educational level**	
Below primary Primary Secondary Tertiary	15 (3.9) 50 (12.9) 91 (23.4) 233 (59.9)
**Employment status**	
Full-time Part-time Unemployed Retired Housewife Student	191 (49.1) 36 (9.3) 6 (1.5) 49 (12.6) 25 (6.4) 82 (21.1)
**Monthly household income**	
HK$10,000 or below HK$10,001 – HK$20,000 HK$20,001 – HK$30,000 HK$30,001 – HK$40,000 HK$40,001 – HK$50,000 Above HK$50,000	75 (19.3) 74 (19.0) 75 (19.3) 73 (18.8) 46 (11.8) 46 (11.8)
**Dose(s) of the COVID-19 vaccine received**	
0 1 2 3 4	5 (1.3) 4 (1.0) 78 (20.1) 275 (70.7) 27 (6.9)
**Type of the COVID-19 vaccine received (*N*** **= 384)**	
BioNTech Sinovac Other	273 (71.1) 110 (28.6) 1 (0.3)

Abbreviations: COVID-19, coronavirus disease 2019; n, number

### CAS

Table 2 presents the C-19ASS results of the participants. The one-sample Kolmogorov–Smirnov test indicated that the C-19ASS-P (D(389) = 0.087; *p* < 0.001) and C-19ASS-A (D(389) = 0.132; *p* < 0.001) scores were not distributed normally. The median score on the C-19ASS-P subscale (items 2, 4, 6, 7, 8, and 9) was 8 (IQR 5–13). Relatively high median scores were obtained for item 2 (‘I have checked myself for the symptoms of COVID-19’; median score: 2 (IQR 1–3)), 7 (‘I have checked my family members and loved one for the symptoms of COVID-19’; median score: 2 (IQR 1–3)), and 9 (‘I have imagined what could happen to my family members if they contracted COVID-19’; median score: 2 (IQR 1–3)). Conversely, a low median score was obtained for item 6 (‘I have read about news relating to COVID-19 at the cost of engaging in work’; median score: 0 (IQR 0–1)). The median score on the C-19ASS-A subscale (item 1, 3, and 5) was 3 (IQR 0–6). A relatively high median score was obtained for item 5 (‘I have avoided touching things in public spaces because of the fear of contracting COVID-19’; median score: 2 (IQR 0–3)).

**Table 2:** COVID-19 anxiety syndrome scale among the participants (*N* = 389)

Items	Median (IQR)
1. I have avoided using public transport because of the fear of contracting coronavirus (COVID-19).	1 (0–2)
2. I have checked myself for symptoms of coronavirus (COVID-19).	2 (1–3)
3. I have avoided going out to public places (shops, parks) because of the fear of contracting coronavirus (COVID-19).	1 (0–2)
4. I have been concerned about not having adhered strictly to social distancing guidelines for coronavirus (COVID-19).	1 (0–2)
5. I have avoided touching things in public spaces because of the fear of contracting coronavirus (COVID-19).	2 (0–3)
6. I have read about news relating to coronavirus (COVID-19) at the cost of engaging in work (such as writing emails, and working on word documents or spreadsheets).	0 (0–1)
7. I have checked my family members and loved one for the signs of coronavirus (COVID-19).	2 (1–3)
8. I have been paying close attention to others displaying possible symptoms of coronavirus (COVID-19).	1 (1–3)
9. I have imagined what could happen to my family members if they contracted coronavirus (COVID-19).	2 (1–3)

Abbreviations: COVID-19, coronavirus disease 2019; IQR, interquartile range. Note: COVID-19 Anxiety Syndrome Scale - Perseveration: Items 2, 4, 6, 7, 8, and 9. COVID-19 Anxiety Syndrome Scale - Avoidance: Items 1, 3, and 5

### CVH

Table 3 presents the CVHS results of the participants with CVH and those without CVH. The prevalence of CVH was 68.1%, as estimated on the basis of the participants’ CVHS scores. A significantly larger proportion of participants with CVH rated “hesitant” compared with those without CVH across all CVHS items (*p* < 0.001). Specifically, participants with CVH were more reluctant to receive the nationally recommended COVID-19 vaccine than were participants without CVH (participants with CVH: 89.1%; participants without CVH: 24.2%; χ2(2) = 172.402; *p* < 0.001). Participants with CVH were also more concerned about vaccine safety (participants with CVH: 91.3%; participants without CVH: 29.8%; χ2(2) = 164.364; *p* < 0.001) and about potential vaccine side effects (participants with CVH: 91.7%; participants without CVH: 39.5%; χ2(2) = 131.899; *p* < 0.001) than were participants without CVH. Participants with CVH were more likely to deem it unnecessary to receive a COVID-19 vaccine at present (participants with CVH: 74.0%; participants without CVH: 20.2%; χ2(2) = 116.761; *p* < 0.001) and to prefer receiving fewer vaccines (participants with CVH: 76.6%; participants without CVH: 37.9%; χ2(2) = 60.359; *p* < 0.001) than were those without CVH. Furthermore, participants with CVH were more likely to be concerned about vaccine efficacy (participants with CVH: 77.7%; participants without CVH: 20.2%; χ2(2) = 133.440; *p* < 0.001) and to prefer acquiring immunity through COVID-19 infection than through vaccination (participants with CVH: 60.8%; participants without CVH: 25.0%; χ2(2) = 43.503; *p* < 0.001) than were those without CVH.

**Table 3:** COVID-19 vaccine hesitancy scale among the participants with or without COVID-19 vaccine hesitancy (*N* = 389)

Items	With COVID-19 vaccine hesitancy (*N* = 265)			Without COVID-19 vaccine hesitancy (*N* = 124)			Chi-square test
	*n* (%)			*n* (%)			
	Hesitant	Not sure or not know	Not hesitant	Hesitant	Not sure or not know	Not hesitant	
1. Would you delay getting the COVID-19 vaccine for reasons other than illness or allergy?	194 (73.2)	22 (8.3)	49 (18.5)	19 (15.3)	17 (13.7)	88 (71.0)	χ2(2) = 120.208, *p* < 0.001
2. Would you decide not to get the COVID-19 vaccine for reasons other than illness or allergy?	166 (62.6)	32 (12.1)	67 (25.3)	8 (6.5)	13 (10.5)	103 (83.1)	χ2(2) = 124.346, *p* < 0.001
3. Would you want to get the nationally recommended COVID-19 vaccine?	236 (89.1)	20 (7.5)	9 (3.4)	30 (24.2)	34 (27.4)	60 (48.4)	χ2(2) = 172.402, *p* < 0.001
4. It is not necessary to get the COVID-19 vaccine at this time?	196 (74.0)	31 (11.7)	38 (14.3)	25 (20.2)	17 (13.7)	82 (66.1)	χ2(2) = 116.761, *p* < 0.001
5. I believe that the COVID-19 vaccine can prevent COVID-19 (COVID-19 is a severe disease).	131 (49.4)	59 (22.3)	75 (28.3)	9 (7.3)	13 (10.5)	102 (82.3)	χ2(2) = 102.132, *p* < 0.001
6. It is better for you to develop immunity by getting COVID-19 than to get the COVID-19 vaccine.	161 (60.8)	63 (23.8)	41 (15.5)	31 (25.0)	53 (42.7)	40 (32.3)	χ2(2) = 43.503, *p* < 0.001
7. It is better for you to get fewer vaccines at the same time.	203 (76.6)	47 (17.7)	15 (5.7)	47 (37.9)	45 (36.3)	32 (25.8)	χ2(2) = 60.359, *p* < 0.001
8. How concerned are you that you might have a serious side effect from getting the COVID-19 vaccine?	243 (91.7)	12 (4.5)	10 (3.8)	49 (39.5)	14 (11.3)	61 (49.2)	χ2(2) = 131.899, *p* < 0.001
9. How concerned are you that the COVID-19 vaccine might not be safe?	242 (91.3)	12 (4.5)	11 (4.2)	37 (29.8)	18 (14.5)	69 (55.6)	χ2(2) = 164.364, *p* < 0.001
10. How concerned are you that a shot might not prevent COVID-19?	206 (77.7)	33 (12.5)	26 (9.8)	25 (20.2)	24 (19.4)	75 (60.5)	χ2(2) = 133.440, *p* < 0.001
11. Would you recommend your friends and family members to get the COVID-19 vaccine?	168 (63.4)	56 (21.1)	41 (15.5)	9 (7.3)	14 (11.3)	101 (81.5)	χ2(2) = 163.794, *p* < 0.001
12. Overall, how hesitant about the COVID-19 vaccine would you consider yourself to be?	219 (82.6)	26 (9.8)	20 (7.5)	12 (9.7)	16 (12.9)	96 (77.4)	χ2(2) = 214.778, *p* < 0.001
13. I trust the information I receive about the COVID-19 vaccine.	143 (54.0)	67 (25.3)	55 (20.8)	11 (8.9)	8 (6.5)	105 (84.7)	χ2(2) = 142.840, *p* < 0.001
14. I am able to openly discuss my concerns about shots of the COVID-19 vaccine with the doctor.	63 (23.8)	61 (23.0)	141 (53.2)	2 (1.6)	5 (4.0)	117 (94.4)	χ2(2) = 64.339, *p* < 0.001
15. All things considered, how much do you trust the doctor?	105 (39.6)	84 (31.7)	76 (28.7)	6 (4.8)	26 (21.0)	92 (74.2)	χ2(2) = 79.776, *p* < 0.001

Abbreviations: COVID-19, coronavirus disease 2019; n, number

### Association between CAS and CVH

The median C-19ASS-P score was significantly higher for participants without CVH than for those with CVH (participants without CVH: 10 (IQR 6–15); participants with CVH: 8 (IQR 4–12); U = 12853.500, Z = -3.466; *p* = 0.001). Similarly, the median C-19ASS-A score was significantly higher for participants without CVH than for those with CVH (participants without CVH: 5 (IQR 2–8); participants with CVH: 3 (IQR 0–5); U = 12153.500, Z = -4.183; *p* < 0.001).

## Discussion

Our findings reveal an association between CAS and CVH, shedding light on the intricate interplay between psychological factors and vaccination attitudes. As the world grapples with the persisting challenges of the COVID-19 pandemic, holistic interventions addressing both informational and psychological aspects are needed to increase the rate of vaccine acceptance and to mitigate the effects of CAS on public health and mental health outcomes [39, 40].

The evaluation of CAS provides insights into the psychological aspects of individuals’ experiences during the pandemic [41, 42]. Our study indicates that CAS persists in the postpandemic era, although its level is lower than that noted in the acute phase of the pandemic [11]. Elevated median scores on specific items of the C-19ASS, such as those related to self-monitoring for symptoms, checking on family members, contemplating potential consequences, and avoiding public places, highlight the pervasive nature of anxiety-related behaviours and the effects of pandemic-related stress on daily functioning. These findings are consistent with the broader understandings of anxiety disorders, which emphasises the transdiagnostic features of threat overestimation and intolerance to uncertainty.

Our study revealed a high prevalence of CVH. Participants with CVH were more reluctance towards receiving the nationally recommended vaccine because of elevated concerns regarding safety, side effects, perceived vaccination need, and actual vaccine efficacy. This underscores the multifaceted nature of CVH, which extends beyond logistical or informational barriers to encompass deeply ingrained anxieties pertaining to COVID-19 vaccines [43].

An association has been identified between COVID-19-related anxiety and vaccine acceptance [26]. Our study offers evidence from a different perspective, indicating an association between CAS and CVH. Participants with CVH had relatively low mean scores on both the C-19ASS-P and C-19ASS-A subscales. The finding suggests an association wherein individuals with CVH are less likely to adopt maladaptive coping strategies in response to the threat and fear of COVID-19 and that individuals with lower levels of CAS are more likely to develop CVH. Approximately 60.8% of the participants with CVH reported believing that they should build immunity through COVID-19 infection rather than through vaccination. This belief may stem from a reduced fear of COVID-19 or underestimation of its health implications [44]. Individuals who received COVID-19 vaccines were primarily motivated by a desire for protection against COVID-19 [45]. Individuals who were reluctant to receive a COVID-19 vaccine may have judged the risk of contracting COVID-19 to be lower than the risk of vaccine side effects. Consequently, individuals with CVH may exhibit lower levels of CAS: they perceive the threat as minor and thus have low scores on the C-19ASS-P and C-19ASS-A subscales [9].

Individuals without CVH might have experienced considerable emotional distress during the early stages of the pandemic [7, 8]. The distress and ongoing threat of COVID-19 might have induced CAS in these individuals. The low CVH level indicates their efforts to cope with COVID-19-related anxiety. Because psychological distress can lead to perseveration in people in response to COVID-19, individuals exposed to such distress are less hesitant to take measures (i.e., vaccination).

Our findings regarding the association between CAS and CVH hold major implications for public health strategies. Given the association between CAS and CVH, public health campaigns for disseminating credible information on COVID-19 should be introduced to reduce hesitancy and scepticism towards vaccination [26]. Such campaigns may benefit individuals with maladaptive coping strategies by providing them with an opportunity to regain a sense of control over their health and helping them address their anxiety driven by perceived threats. Accurate information can reduce uncertainty regarding the risk or reliability of vaccination and counter conspiracy theories [23, 44], thus alleviating CVH. Individuals with high CAS levels may exhibit low CVH levels. Accurate information facilitates cognitive reappraisal [46] and alleviates COVID-19-related perseveration and avoidance behaviours by assuring individuals of vaccine-conferred health protection.

In the postpandemic era, most residents of Hong Kong received two or three doses of COVID-19 vaccines. Adaptive coping strategies in response to the threat and fear of COVID-19 and the inclination to avoid public places may be influenced by social norms and acquaintance behaviours. If most residents and their family members and peers have been vaccinated, it can create a social influence that reduces CVH. Despite having high CVH levels, some individuals may normalize their maladaptive coping strategies by undergoing vaccination. However, individual responses to the threat and fear of COVID-19 are complex and multifaceted, influenced by factors such as personal experiences, cultural background, and interpersonal differences [26]. Notably, the aforesaid explanations may not be applicable to every individual with CAS or CVH. Thus, caution should be exercised when interpreting the findings.

CAS, a psychopathology, results from the adoption of maladaptive coping strategies in response to perceived threats and is exacerbated by uncertainties and safety concerns introduced by the pandemic. Anxiety or mood disorders can intensify COVID-19-related stress, which explains the disproportionate effects of CAS on individuals with existing mental health conditions [47]. Our findings revealed that maladaptive coping in response to COVID-19 and avoidance behaviours pertaining to COVID-19 transmission can protect individuals from becoming trapped in a state of persistent distress and help them return to normal functioning. The need for predictability, excessive information seeking tendency, and decision-making difficulties associated with intolerance to uncertainty, substantially increases the level of CAS.

Understanding the interplay between CAS and CVH is essential for developing targeted public health interventions. Strategies must extend beyond addressing factual concerns about vaccine safety and efficacy to include mitigating psychological barriers rooted in anxiety. CAS-associated compulsive checking behaviours and information-seeking tendencies must be addressed to promote vaccine acceptance.

This study clarified the association between CAS and CVH in an ethnic Chinese population. Our findings may guide healthcare authorities, policymakers, and public health practitioners in devising strategies and policies for mitigating CVH and managing COVID-19-related maladaptive coping behaviours, thereby reducing psychological distress and helping individuals return to normal functioning.

### Strengths and limitations of the study

The study is the first to investigate the association between CAS and CVH in the general population in the postpandemic era. We used the structured and well-validated C-19ASS to evaluate the level of CAS and the comprehensive and well-validated CVHS to determine the presence of CVH. Furthermore, the sample size was sufficiently large; sampling was performed using both online and in-person methods to enhance representativeness.

Our study has several limitations. Its cross-sectional nature prohibited causal inferences. Despite using both online and in-person methods for sampling, approximately 54.5% of the participants were aged 18–35 years, and 80% were educated at tertiarily and secondary levels; Therefore, generalizability may be limited, particularly among populations with lower educational attainment, which could lead to a weaker association due to reduced awareness of infectious diseases and their effects on health behaviours. Furthermore, because a self-report questionnaire was used for data collection, the possibilities of recall and social desirability biases cannot be ignored.

## Conclusion

This study confirms the persistence of CAS in the postpandemic era and indicates an association between CAS and CVH. Our findings underscore the need for comprehensive interventions aimed at addressing both informational and psychological aspects to increase the rate of vaccine acceptance and mitigate the effect of CAS on public health outcomes. Information on CAS and vaccine-related concerns can guide personalised public health intervention for promoting vaccination and facilitating adaptive coping. Such interventions can facilitate a return to normal functioning.

## Electronic supplementary material

Below is the link to the electronic supplementary material.

Supplementary Material 1

## Data Availability

Data supporting the findings of the present study are available from the corresponding author upon reasonable request.
